# Humoral responses to HIVconsv induced by heterologous vaccine modalities in rhesus macaques

**DOI:** 10.1002/iid3.52

**Published:** 2015-03-11

**Authors:** Nicola J Borthwick, Maximillian Rosario, Torben Schiffner, Emma Bowles, Tina Ahmed, Peter Liljeström, Guillaume E Stewart-Jones, Jan W Drijfhout, Cornelis J M Melief, Tomáš Hanke

**Affiliations:** 1 Nuffield Department of Medicine, The Jenner Institute, University of Oxford, Old Road Campus Research Building, Roosevelt DriveOxford, OX3 7DQ, UK; 2 Nuffield Department of Medicine, MRC Human Immunology Unit, Weatherall Institute of Molecular Medicine, University of Oxford, The John RadcliffeOxford, OX3 9DS, UK; 3 Nuffield Department of Medicine, The Sir William Dunn School of Pathology, University of Oxford, South Parks RoadOxford, OX1 3RE, UK; 4 Department of Microbiology, Tumor and Cell Biology, Karolinska InstitutetStockholm, Sweden; 5 Departement of Immunohematology and Blood Transfusion, Leiden University Medical CentreLeiden, the Netherlands

**Keywords:** Antibodies, conserved regions, HIV vaccines, macaques, synthetic long peptides

## Abstract

Vaccines delivering T cell immunogen HIVconsv vectored by plasmid DNA, non-replicating simian adenovirus and non-replicating modified vaccinia virus Ankara (MVA) are under clinical evaluation in phase I/IIa trials in UK, Europe, and Africa. While these vaccines aim to induce effector T cell responses specific for HIV-1, we here characterized the humoral responses induced by HIVconsv administration to macaques using six different vaccine modalities: plasmid DNA, human adenovirus serotype 5, simian adenovirus serotype 63, MVA, Semliki Forest virus replicons, and adjuvanted overlapping synthetic long peptides (SLP). We found that only the SLP formulation, but none of the genetic vaccine platforms induced antibodies recognizing linear HIVconsv epitopes, median 32/46 SLP.HIVconsv peptides. These antibodies bound to 15-mer and SLP peptides, recombinant gp120 and trimeric gp140 of HIV-1 Bal, YU2, JRFL, and UG037, but failed to react with HIV-1 Bal and IIIB virions and HIV-1 Bal- and IIIB-infected human cells, and consequently failed to induce neutralizing antibodies. The HIVconsv immunogen contains conserved regions derived from Gag, Pol, Vif, and Env proteins of HIV-1, and antibodies induced by the SLP.HIVconsv vaccination resulted in positive signals in routine HIV-1 tests. Thus, only HIVconsv delivered by SLP resulted in seroconversion, an observation that provides important guidance for recruiting volunteers into future clinical trials. Furthermore, our data confirms that vaccine delivery by SLP induces humoral as well as cellular immune responses and could be considered for inclusion in future vaccine regimens where this is required.

## Introduction

Even against the background of antiretroviral treatment and prevention, a vaccine against HIV-1 infection will always be a key to controlling the AIDS epidemic. Ideally, such a vaccine will induce balanced responses mediated by effective killer T cells and beneficial antibodies. Both killer T cells and antibodies will need to recognize diverse global variant strains and escape mutants of HIV-1 1,2.

The specificity of vaccine-elicited responses is dictated by the HIV-1-derived immunogens, while the magnitude, longevity, functionality, and anatomical distribution of the individual components of immune responses are primarily determined by the route and means of the delivery of the subunits to the immune system 3–7. To induce sufficiently robust responses to the transgene product and avoid the build-up of anti-vector antibodies, which stands the risk of decreasing vaccine take 8,9, the HIV-1 subunits are typically delivered by a sequential combination of heterologous vectors in prime-boost regimens 5.

A major challenge facing the development of successful HIV-1 vaccines is the enormous variability of different HIV-1 strains circulating in the population 10. This can be tackled by using cocktails of immunogens or computed artificial amino acid (aa) sequences 11,12. Our strategy has been to focus the vaccine-elicited responses on the conserved regions of the HIV-1 proteome, which are common to many HIV-1 strains and decrease HIV-1 fitness if mutated 13–16. The immunogen HIVconsv was derived from the 14 most conserved regions of the HIV-1 proteome 17. HIVconsv does not contain immunodominant but variable epitopes typically recognized first during natural infection, but rather focuses the immune response on conserved sub-dominant epitopes, which may prove important for protection 18,19.

The gene encoding HIVconsv has been delivered by a range of non-replicating vaccine vectors including plasmid DNA, human and chimpanzee adenoviruses, modified vaccinia Ankara (MVA), and Semliki Forest virus (SFV) replicons and was additionally also formulated as adjuvanted overlapping synthetic long peptides (SLP) 17,20–24. The SLP platform showed great promise for treating early stage cancer patients infected with human papilloma virus 25–27. The primary aim of the HIVconsv vaccines is to induce cytotoxic T cells (CTL) capable of killing HIV-1-infected host cells. Induction of such CTL was demonstrated in various pre-clinical models where SLP.HIVconsv was shown to induce T cell responses that were broader and of a greater magnitude than the genetic vaccines 17,20–24. In contrast, although Env-specific antibodies were detected previously following SLP.HIVconsv administration 23, the induction of HIVconsv humoral responses induced by other delivery platforms has not been thoroughly studied. Such responses are important because HIVconsv includes two conserved regions of Env, and antibodies to these external domains may contribute to mechanisms preventing HIV-1 acquisition 28,29. In addition, antibodies to the HIVconsv immunogen could potentially make vaccine recipients falsely positive in commonly employed first-line HIV-1 infection tests. Here, we characterize in depth the humoral responses elicited by a sequential heterologous administration of the HIVconsv immunogen to rhesus macaques.

## Materials and Methods

### Genetic vaccine preparations

To prepare pTH.HIVconsv, the synthetic gene coding for HIVconsv (GeneArt) was subcloned into plasmid pTH. The plasmid DNA for immunizations was prepared using the Endo-Free Gigaprep (Qiagen) and stored at −20°C until use. For HAdV-5.HIVconsv, recombinant adenovirus was obtained using the AdEasy™ Adeno Viral Vector System (Stratagene) following the manufacturer's instructions. To prepare recombinant MVA, chicken embryo fibroblasts (CEF) were infected with parental MVA and then transfected using Superfectin (Qiagen) with pSC11.HIVconsv. The MVA.HIVconsv was subjected to five rounds of plaque purification and the masterstock purified on a 36% sucrose cushion, titered and stored at −80°C until use. The primary Chimpanzee adenovirus 63 was amplified on HEK293 cells and the virus genome modified by deleting E1 and E3 and substituting the native E4 region with HAdV-5 ORF 6. The expression cassette was inserted into the E1 region by homologous recombination in *E. coli* strain 5183. The ChAdV63.HIVconsv virus was rescued in a HEK293 derived line expressing the tet repressor by transfecting the pChAdV63.HIVconsv pre-Adeno plasmid DNA and further amplified by serial passaging. The virus was purified by two CsCl gradient centrifugations, titered and stored at −80°C. For the preparation of VREP.HIVconsv the HIVconsv ORF was inserted into plasmid pSFVb12a, which attached a 34 aa enhancer sequence of the capsid and the foot-and-mouth disease virus 2a cleavage site into the HIVconsv gene. A two-helper RNA system was used to package the HIVconsv RNA into the recombinant SFV particles. The virus was purified and indirect immunofluorescence of infected BHK cells used to determine the concentration.

### Animals and procedures

Young adult male, 30- to 36-month old, Indian rhesus macaques (*Macaca mulatta*) designated One, Ozone, and Octavia bred at the Centre for Macaques, Porton Down, UK were used. Blood was drawn from superficial veins prior to, during and after vaccine administration. All animal procedures and care strictly conformed to UK Home Office Guidelines under PPL no. 30/2424 held by Professor Sir Andrew McMichael of the University of Oxford, on which T.H. was the deputy holder. Experiments were conducted in the spirit of the National Centre for the Replacement, Refinement and Reduction of Animals in Research.

### Plasma isolation

For ELISA, plasma was isolated from peripheral blood, clarified by centrifugation, aliquoted and stored at −20°C until use. For selected samples, IgG was purified from plasma using Nab™ protein G spin columns (Thermo Scientific, Waltham, MA) following the manufacturer's instructions. Fractions were combined and concentrated using an Amicon Ultra-15 centrifugal filter unit with a 30,000 molecular mass cut-off (Millipore, Watford, UK). The concentration of IgG was measured using NanoDrop (Thermo Scientific).

### Peptides

**Figure 1:**
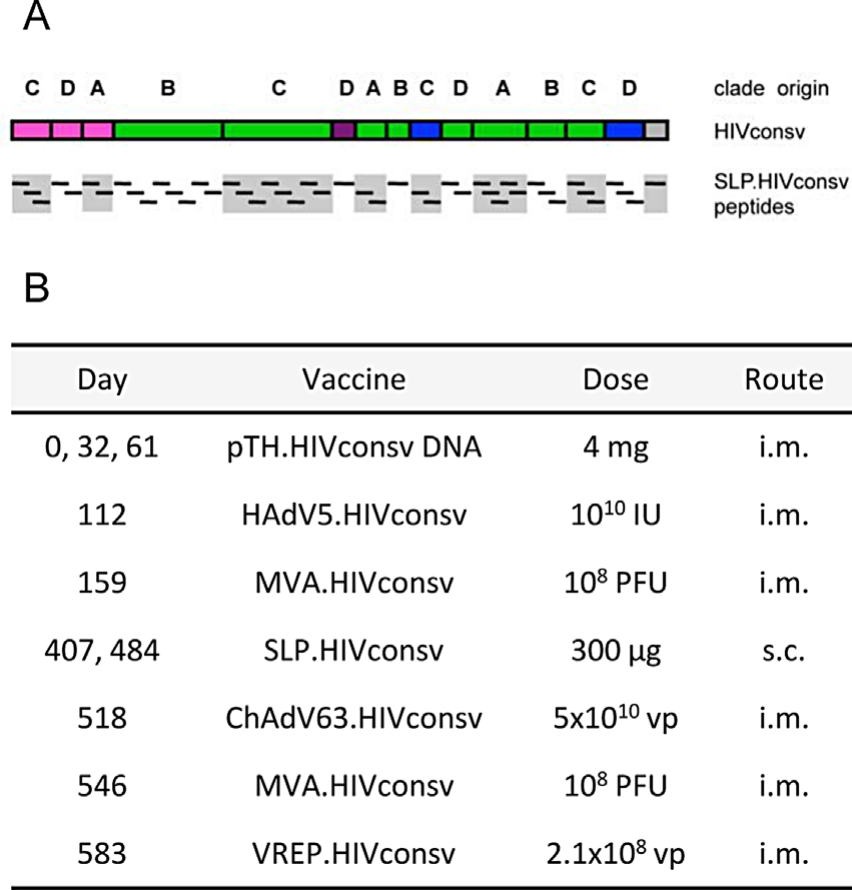
(A) Schematic representations of the HIVconsv immunogen and the 46 peptides of the SLP.HIVconsv vaccine. The gray boxes emphasize the fact that the SLP do not go across region junctions. The HIV-1 proteins of origin are colour-coded: pink—Gag, green—Pol, blue—Env, and purple—vif. (B) Summary of the immunization regimen. IU—infectious units; PFU—plaque-forming units; vp—virus particles; i.m.—intramuscular; and s.c.—subcutaneous.

Two sets of peptides were used. The first set was the actual SLP.HIVconsv vaccine and was composed of 46 synthetic peptides 25–28 aa in length that overlap by 11 aa. These peptides were synthesized at the Department of Clinical Pharmacy and Toxicology, Leiden University Medical Centre, Leiden, the Netherlands and span across each of the 14 regions of the HIVconsv immunogen stopping at the region junctions (Fig. 1A). The second set of peptides were 15-mers overlapping by 11 aa (15/11) (Sigma–Aldrich, Dorset, UK), which were previously used to analyze T cell responses in an interferon (IFN)-γ enzyme-linked immunospot (ELISPOT) assay. These peptides were >80% pure by mass spectroscopy, span the entire HIVconsv protein including junctions and were generously provided by the International AIDS Vaccine Initiative. Both the SLP and 15/11 peptides were reconstituted to 40 mg/mL in dimethyl sulfoxide (DMSO), diluted to 100 μg/mL in PBS and stored at −80°C until use. For some assays, peptides were combined into six pools consisting of 32–35 peptides each at a concentration of 15 μg/mL in PBS.

### Recombinant Env proteins

Recombinant HIV-1 gp120 and gp140 glycoproteins derived from HIV-1 isolates BaL (AY713409, clade B), JRFL (U63632, clade B), UG037 (AB253426, clade A), and YU2 (EF589040, clade A) were produced by transient transfection of HEK 293T cells and subsequent purification as previously described 30.

### Monoclonal antibodies

Human monoclonal antibodies (mAbs) F240 31 and 2G12 32 were obtained through the NIH AIDS Research & Reference Reagent Program (NIH AIDS RRRP). Human mAb VRC01 33 and 10E8 34 were transiently expressed in HEK 293T cells under serum-free conditions, purified by protein A affinity chromatography and their purity and integrity were verified by SDS-PAGE and ELISA, respectively.

### ELISA

ELISA plates (BD Biosciences, Oxford, UK) were coated with peptide pools at 0.5 µg/mL, or individual peptides or recombinant proteins at 1 μg/mL in carbonate/bicarbonate buffer at 4°C overnight. After washing 5X with PBS, 0.2% Tween 20 (PBSt; Sigma–Aldrich, Dorset, UK), the plates were blocked with protein-free blocking buffer (Thermo Scientific) for 1 h at room temperature (RT). Plasma samples diluted in PBSt were added at RT for 2 h, washed and bound antibodies were detected either using goat anti-rhesus IgG-alkaline phosphatase (ALP; Southern-Biotech, USA) or goat anti-human IgM-ALP (Sigma–Aldrich) at RT for 1 h. The plates were washed 5X with PBSt, p-nitrophenylphosphate substrate (Sigma–Aldrich) was added at RT for 45 min, the reaction was stopped with the addition of 25 μl 3 N NaCl (Sigma–Aldrich) and the plates read at 405 nm using a μQuant ELISA plate reader (Biotek Instruments, Potton, UK).

Positive and negative controls were run on each plate. Negative controls were wells containing no plasma, pre-immune plasma and an irrelevant 15-mer peptide (NAQGQMHQALSPRTL) not found in HIVconsv. The HIVconsv protein contains a C-terminal tag Pk recognized by murine mAb; pooled plasma samples from HIVconsv immunized animals at 1:100 dilution were used as a positive control together with a 14-mer peptide RAFVT**IPNPLLGLD** containing the murine mAb epitope (bold). Results are shown either as log_10_ titre based on the pre-immune control sample at 1:50 dilution ± 2 SD's or as ELISA units (EU) in the mapping studies, whereby the response of the test sample is shown as a percentage of the positive control 35.

### HIV tests

Plasma samples from vaccinated animals were tested for HIV-1 positivity by the John Radcliffe Hospital, Department of Microbiology & Infectious Diseases using a high throughput Alere Determine™ HIV-1/2 Ag/Ab Combo assay (Alere Technologies, Stirling UK). The plasma samples were further investigated using another routine ELISA-based assay, anti-HIV TETRA (Bio-Rad, Hemel Hempstead, UK), which is a sandwich ELISA that utilizes a plate pre-coated with three recombinant HIV proteins gp41, gp36, and p24 and one p24 peptide together with rabbit control sera and it is not species specific. The New LAV Blot I kit (Bio-Rad) for anti-HIV-1 antibody detection in serum/plasma by immunoblotting was used for confirmation of HIV-1 positivity in the plasma samples.

### HIV-1 capture assay

An ELISA-based HIV-1 capture assay was used to determine if IgG from vaccinated animals could bind to free HIV-1 36. Two laboratory strains of HIV-1 were used in the capture assay, HIV-1 Bal (AY713409, clade B, obtained from the Centre for AIDS Reagents, NIBSC and donated by Drs S Gartner, M Popovic, and R Gallo, courtesy of the NIH AIDS RRRP) and a Nef mutated HIV-1 IIIB (K03455, clade B, a kind gift from Dr Peter Hayes, IAVI Human Immunology Laboratory, London). Both HIV-1-stocks were passaged on human CD4^+^ lymphocytes and the MOI and infection rates determined as previously described 37,38. ELISA plates were coated at 4°C overnight with goat anti-human IgG Fc (Southern–Biotech, Birmingham, Alabama, USA) at 1 µg/mL in carbonate/bicarbonate buffer and blocked with blocking buffer as described for ELISA's except that PBS was used for all washes and as a diluent. Rhesus IgG purified from plasma was added at 0.1–20 μg/mL and plates incubated at RT for 1 h. The negative control, normal human IgG and the human monoclonals (F240, 2G12, VRC01, and 10E8) were all used at 1 μg/mL. Empty binding sites were blocked for at RT 1 h using normal human serum (Sigma–Aldrich) diluted to 1 in 5,000. Each stain of HIV-1 was adjusted to 100–500 ng/mL by p24 antigen content and 50 μL added to each well. After 1 h incubation at 37°C, unbound virus was washed off and bound virus quantified using an HIV-1 p24 antigen ELISA (ZeptoMetrix Corp.) following the manufacturers’ instructions.

### Reactivity with HIV-1-infected cells

Flow cytometry was used to determine if IgG from vaccinated animals could bind to virally infected cells. Human CD4^+^ T cells were infected with HIV-1 Bal or IIIB as previously described 39. Briefly, CD4^+^ T cells purified from PBMC by MACS (Miltenyi Biotec, Surrey, UK) depletion were activated with PHA for 3 days and then infected with HIV-1 at an MOI of 0.005–0.01 by spinoculation. Infected cells were cultured for 5 days, washed, adjusted to 2 × 10^6^/mL in PBSA (PBS, 0.2% BSA and 0.02% sodium azide), stained in round bottom 96-well plates with a viability dye (Live/Dead®, Molecular Probes, Life Technologies) to exclude dead cells, and incubated with the human mAbs 2G12, VRC01, and F240 at 1.0 μg/mL and 10 μg/mL, or with IgG from vaccinated animals at 10 μg/mL and 50 μg/mL at 4°C for 30 min. Plates then were washed 2X with PBSA and the bound antibody was visualized with the addition of FITC- or PE-conjugated secondary Ab to human and rhesus IgG (Southern–Biotech). Controls were uninfected cells, cells without the primary antibody and IgG purified from pre-immune plasma. The cells were acquired on an LSR II flow cytometer (Becton–Dickinson, UK) and the data were analyzed using Flowjo.

### HIV-1 neutralization assay

The ability of antibody to neutralize HIV-1 was assessed using the TZM-bl assay with molecularly cloned pseudoviruses MN.3, MW965.26, TH023.6, and MLV-SVA. Pseudovirus was incubated with serially diluted antibody for 1 h at 37°C before plating out with TZM-bl cells. After a 48-h incubation at 37°C, the cells were lysed, and luciferase signal in the lysate was developed with Britelite Plus substrate (1:1, v/v; PerkinElmer Life Sciences) and read in a luminometer 40.

## Results

### Vaccines

The HIVconsv immunogen was designed to contain only those regions of HIV-1 that are conserved across the major clades. The largest component is Pol followed by Gag and it also contains smaller regions of Env and Vif. (Fig. 1A) A total of six vaccine modalities were used to deliver the HIVconsv to rhesus macaques: pTH.HIVconsv, non-replicating HAdV5.HIVconsv, non-replicating MVA.HIVconsv, SLP.HIVconsv emulsified in an equal volume of Montanide ISA-51 and injected 24 h after topical application of Imiquimod (Aldara, 3 M), non-replicating ChAdV63.HIVconsv and VREP.HIVconsv in a sequential regimen (Fig. 1B).

### SLP, but not genetic vaccines, induce HIVconsv-specific Abs

**Figure 2:**
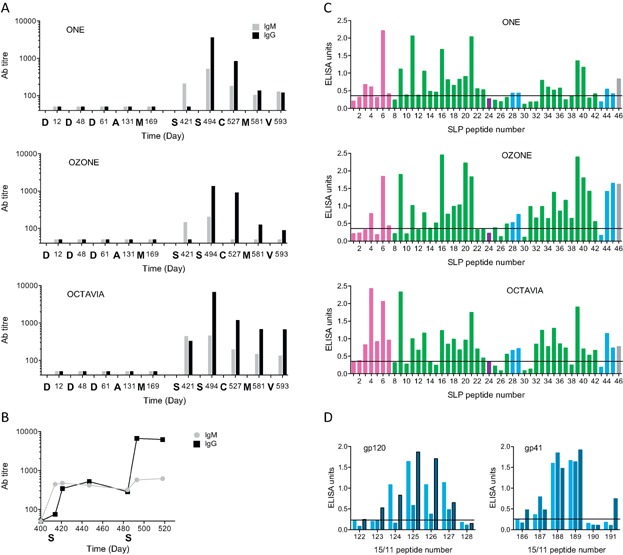
Antibody responses to HIVconsv-derived 15-mer and SLP peptides. (A) IgM and IgG responses to 15/11 peptides throughout the entire experiment. Vaccine administrations are indicated below the graphs as D— pTH.HIVconsv DNA; A—HAdV5.HIVconsv; M—MVA.HIVconsv, S—SLP.HIVconsv; C—ChAdV63.HIVconsv and V—VREP.HIVconsv. (B) IgM and IgG responses using more frequent bleeding time points to capture the primary IgM and secondary IgG Ab responses following SLP.HIVconsv administration. (C) Quantifying Ab responses to individual long peptides of the SLP.HIVconsv vaccine. The HIV-1 proteins of origin are colour-coded: pink—Gag, green—Pol, blue—Env, and purple—Vif. The horizontal lines denote the cut off of the Ab response defined by the activity of pre-immune plasma in the same assay. (D) Quantifying Ab responses to the two HIVconsv Env regions using 15/11 overlapping peptides. Three shades of blues from left to right correspond to animals One, Ozone, and Octavia.

Blood was drawn regularly throughout the study. Initially pre-immune plasma samples and plasma taken 7–10 days following each immunization were assessed for titres of HIVconsv-specific antibodies (Ab) using six pools of 15/11 peptides spanning the entire HIVconsv protein in a standard ELISA. The kinetics and magnitude of the responses observed for each of the six peptide pools were similar and indicated that three immunizations with pTH.HIVconsv DNA followed by HAdV5.HIVconsv and MVA.HIVconsv did not induce IgM or IgG titres above the control, pre-immune levels (Fig. 2A). In all three animals, Ab responses were only induced by the SLP.HIVconsv immunization, peaking after the second administration and thereafter decreased by a factor of approximately 10 over a period of 97 days. There was no evidence of further boosting on subsequent immunization with ChAdV63.HIVconsv, MVA.HIVconsv, or VREP.HIVconsv vaccines (see Supporting Table S1). Additional plasma samples from several time points between the two SLP.HIVconsv immunizations (Fig. 2B) showed that IgM titres appeared earlier and were of a greater magnitude than IgG. This was reversed by the second SLP.HIVconsv delivery, which boosted the IgG but not the IgM response, further confirming that the first and second SLP.HIVconsv delivery triggered typical primary and secondary Ab responses, respectively. Peak IgG antibody responses occurred 10 days after the second SLP.HIVconsv and were maintained at 30 days. This time point was used in all subsequent assays of antibody specificity and function.

### Mapping anti-HIVconsv Ab responses

The SLP.HIVconsv vaccine consists of 46 peptides of 25–28 aa in length (Fig. 1A). Using plasma from the peak time point, IgG titres were determined to each of the 46 long peptides, revealing overall similar, but in detail distinct patterns of peptide recognition among the individual animals (Fig. 2C) with a median of 32 (70%) peptides being recognized. Because of their potentially protective responses , two conserved regions of Env, one derived from the gp120 and one from the gp41 domains, were further mapped using the 15/11 peptides. These data show antibody responses to be largely directed against the sequences WKNDMVDQMHEDIISLWDQSLKPCVKL of gp120 and RQLLSGIVQQQNNLLRAIEAQQHL of gp41 (Fig. 2D).

### HIVconsv-induced Abs bind recombinant HIV-1 Env glycoproteins

**Figure 3:**
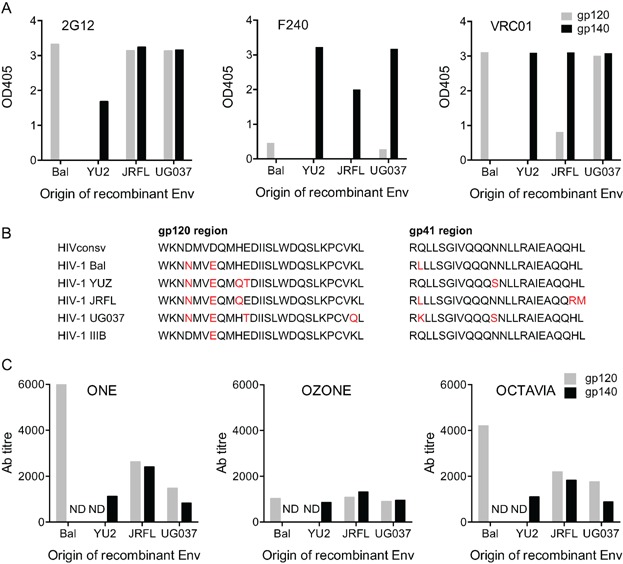
Recognition of recombinant gp120 and gp140 derived from four HIV-1 variants. (A) Characterization of recombinant glycoproteins of origin shown below the graphs by human monoclonal Abs 2G12, F240, and VRC01. Data shown are OD at 405 nm using 1 μg/mL of antibody. (B) Comparison of the aa sequences of gp120 and gp140 mapped in the SLP.HIVconsv recipients with each of the envelope proteins used in the ELISA. Amino acid mismatches from the vaccine are shown in red. (C) Plasma from vaccinated macaques at the peak Ab responses was used to determine the titres against recombinant envelope gp120 and gp140 derived from the four indicated HIV-1 isolates. ND—not done as glycoproteins were unavailable.

Next, Abs were assessed for their ability to bind to glycosylated and folded recombinant envelope glycoproteins gp120 and gp140, which were derived from laboratory and clinical isolates of HIV-1 of Bal, YU2, JRFL, and UG037. First, we tested the recognition of these recombinant proteins by broadly neutralizing human monoclonal Abs. 2G12 32, which is a broadly neutralizing antibody that binds a carbohydrate-dependent epitope on gp120, F240 31, which is a non-neutralizing antibody reactive with a broad range of HIV-1 isolates and is specific for the ectodomain of gp41, and VRC01 33, which has broad HIV-1 neutralization and is specific for the CD4 binding site on gp120 41, showed distinct patterns of reactivities (Fig. 3A). No binding was detected using monoclonal Ab 10E8 34, which binds a conserved region of gp41 just adjacent the transmembrane region (data not shown), suggesting that this epitope was absent or inaccessible. The highest and most consistent activity for gp120 was seen with 2G12 and VRC01, while F240 was more reactive with gp140. Despite some minor aa differences between the recombinant proteins and the HIVconsv immunogen (Fig. 3B), purified IgG from the HIVconsv-vaccinated macaques bound to gp120 and gp140 of the four viruses with medium titres (Fig. 3C). Consistent with anti-15/11 peptide titres, Ozone's Abs were the least reactive of the three animals. There was no evidence that either gp140 or gp120 were more or less well recognized by any of the macaques.

### HIVconsv-induced Abs fail to capture HIV-1 Bal and IIIB virions

**Figure 4:**
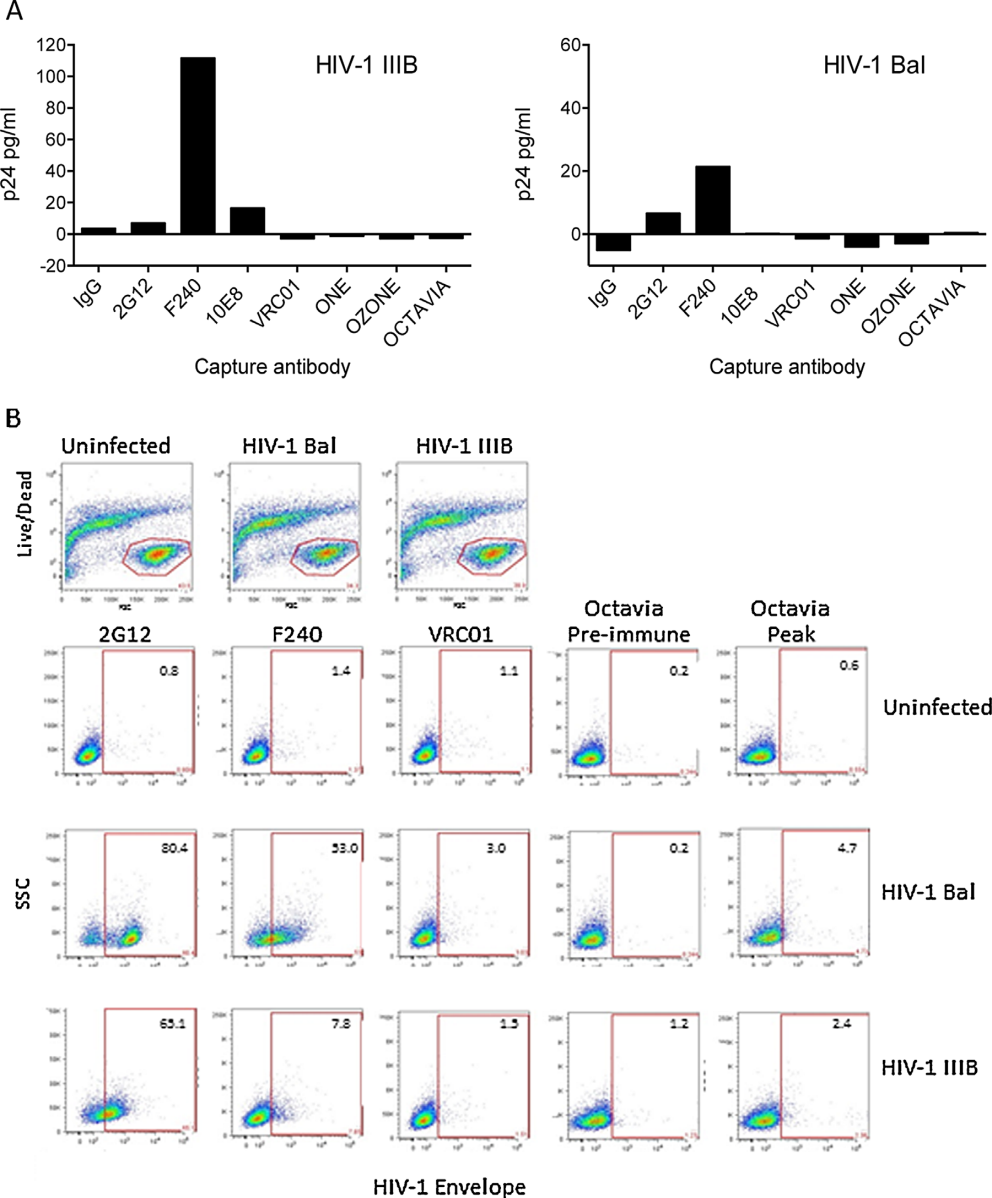
Ab reactivity with virions. (A) HIV-1 capture assay. HIV-1-Bal and IIIB were added at 100 ng/mL and 500 ng/mL, respectively, to pre-coated wells. Purified IgG from vaccinated macaques was used at 25 μg/mL. Irrelevant IgG served as a negative control and determined the zero levels. (B) Recognition of envelope proteins on the surface of HIV-1-infected cells. Human CD4^+^ lymphocytes either uninfected or infected with HIV-1 Bal or HIV-IIIB were investigated for the cell surface expression of envelope proteins using IgG at 10 μg/mL from Octavia at pre-immune and peak Ab response time points. HIV-1 infected cells were also assessed for recognition by three human monoclonal Abs at concentration of 1 μg/mL. Binding was detected using a fluorescence conjugated secondary Ab. Live cells were gated based on cell size and exclusion of a Live/Dead viability dye (Top row). The gates indicate a positive staining as defined by binding of Abs to uninfected cells. Numbers inserted in the top right corner indicate percentages of positive cells.

Encouraged by the binding of the plasma of SLP.HIVconsv-immunized macaques to recombinant glycoproteins, we next tested purified IgG from the same samples for binding activity to free virions of HIV-1 strains Bal and IIIB in a virus capture assay. Irrelevant IgG was used as a negative control and a panel of human monoclonal Abs 2G12, F240, VRC01, and 10E8 was used as positive controls. Of these antibodies, only F240 consistently bound both HIV-1 isolates, while 10E8 and 2G12 mAbs only recognized HIV-1 Bal and IIIB, respectively (Fig. 4A). None of the IgG samples from the vaccinated macaques or the monoclonal Ab VRC01 showed any activity against either of the two HIV-1 isolates (Fig. 4A).

### HIVconsv-induced Abs fail to bind HIV-I Bal and IIIB-infected cells

The potential recognition of Env on the surface of HIV-1-infected cells by purified IgG from vaccinated animals was also investigated by indirect immunofluorescence (Fig. 4B). Human CD4^+^ lymphocytes were infected with HIV-1 strains Bal or IIIB and stained with IgG from pre-immune or vaccinated macaques or with the monoclonal Abs 2G12, F240, or VRC01. Live CD4^+^ T cells were gated based on size and exclusion of a live/dead viability dye. Uninfected cells showed no reactivity with any of the monoclonal or the rhesus antibodies . Cells infected with HIV-1 Bal showed the highest levels of infection and the most clearly defined positive and negative populations when stained using monoclonal Ab 2G12 (Fig. 4B). A slightly lower activity and a poorer definition was found using F240, while VRC01 was virtually negative. A similar pattern was found using HIV-1 IIIB-infected cells although the staining was weaker and the definition poorer. None of the IgG samples from the pre-immune or vaccinated animals showed any activity as illustrated for IgG from Octavia. Pre-immune and peak IgG samples were additionally analyzed for HIV-1 neutralization using the TZM-bl assay against four pseudoviruses MN.3, MW965.26, TH023.6, and MLV-SVA. The results, all given as the sample dilution at which relative fluorescence units (RLS's) were reduced by 50% compared to virus control wells without sample, were all <20 RLU, i.e., there is no detectable virus neutralization compared to positive control sera.

### SLP.HIVconsv vaccination induces seropositivity in HIV-1 tests

**Figure 5:**
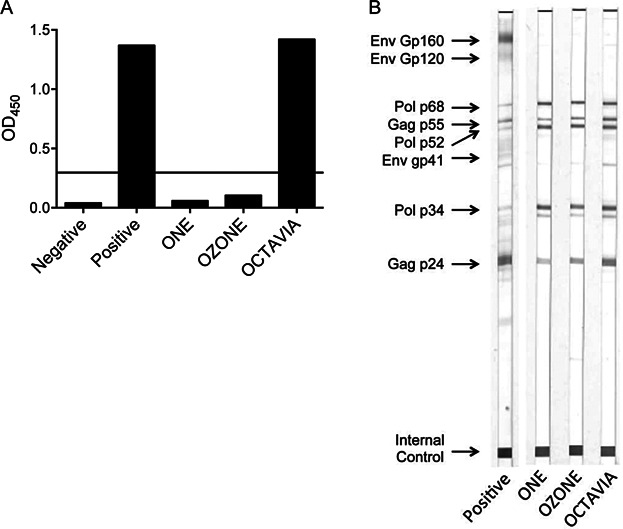
Anti-HIVconsv Ab responses were detected in routine HIV-1 tests. Plasma samples from rhesus macaques at the peak Ab responses were tested using commercial HIV-1 assay kits. (A) The anti-HIV-1 TETRA ELISA. The horizontal line shows the cut off signals for positive responses. (B) New LAV BLOT I confirmation kit for anti-HIV-1 Ab detection in serum/plasma by immunoblotting.

In human trials of candidate HIV-1 vaccines, it is important to know whether vaccination could induce a positive HIV-1 antibody response in routine HIV tests as this has implications on the study design and participant information documentation. To investigate this, plasma from blood samples taken 10 days after the second SLP.HIVconsv immunization, the peak of the response, were sent to a standard pathology laboratory for HIV-1 testing. Using a high throughput Alere Determine™ HIV-1/2 Ag/Ab Combo assay, Octavia was positive and One and Ozone were both borderline (data not shown). Samples were additionally tested using an anti-HIV-1 TETRA ELISA, an assay routinely used to test anti-natal samples in which wells are pre-coated with three recombinant HIV-1 proteins gp41, gp36, and p24 and one p24 peptide. Using this assay, Octavia was again positive and the other two animals were negative (Fig. 5A). Any “borderline” results would normally be sent off for further investigations by Western blotting. Using a commercial Western Blot kit New LAV Blot I, it was clear that antibodies from all three animals recognized a range of HIV-1 proteins (Fig. 5B) and using the kit WHO criteria, all three were categorized as HIV-1 positive.

## Discussion

In the present study, we evaluated the humoral response induced by vaccination with T cell immunogen HIVconsv 17 delivered by six different vaccine modalities. Of these only the SLP, but none of the genetic vectors, induced detectable antibody responses to HIVconsv in all three of the rhesus macaques. This is, perhaps, not surprising given that the HIVconsv immunogen does not contain a leader sequence (Fig. 1A), which would allow the nascent protein to cross the endoplasmic reticulum and be secreted out of the producing cells. Rather the HIVconsv immunogen when expressed from the vaccines within the cells is destined for a relatively rapid degradation in the cytoplasm favoring T cell induction. Note that all vaccine modalities expressed sufficient levels of HIVconsv to elicit T cell responses 24. Thus, primary IgM responses were only observed after the first SLP.HIVconsv immunization with a secondary IgG response of higher magnitude after the second SLP.HIVconsv delivery, which decreased thereafter. This concurs with and expands on our previous report 42.

Other preclinical studies in Aotus monkeys with SLP from *Plasmodium vivax* circumsporozoite antigen in montanide produced strong Ab responses that recognized the peptide immunogen and the native protein on the sporozoites 43. In contrast, antibody responses were not produced in a preclinical study in rabbits using long peptides from papillomavirus E6 and E7 perhaps due to differences in adjuvantation 44. Finally, a phase 1 clinical trial in patients with ovarian cancer using long peptides from the cancer/testis antigen NY-ESO-1 induced an antibody response in 46% of participants 45.

Unlike the humoral response, HIV-1-specific T cell responses were stimulated by all vaccine modalities 21,23,24. Thus, with the adjuvantation used here, the SLP.HIVconsv modality primed and boosted antibody responses and it increased the magnitude and breadth of T cells 23,24, but it was not efficient in priming T cell responses 23.

The HIVconsv proteome contains two highly conserved regions of the envelope glycoprotein of HIV-1. A region of gp120 (HXB2 aa 88–124) forms a part of the V1/V2 stem of the bridging sheet and the *β*1, *α*1, and *β*2 parts of the inner domain, which interacts with gp41 46. Additionally, a region of gp41 (HXB2 aa 11–64) contains part of the fusion protein and the majority of the N-heptad region. In gp41 the N- and C-heptad regions assemble into a stable six-helix bundle structure, a trimer of three N- and three C-heptads, the N-heptads forming the inner core 47. Mapping the antibodies produced by SLP.HIVconsv immunization showed reactivity across the length of the proteome, including both regions of envelope, raising the possibility that HIVconsv induced antibody could have useful anti-viral activity. While the antibody recognition of the recombinant trimers of gp120 and gp140 was encouraging, we were unable to demonstrate any HIV-1 binding activity or virus neutralization. The titres induced by SLP.HIVconsv were quite modest and it may be that a further boost with an additional protein antigen such as recombinant Env would be required to induce detectable anti-virion responses

Antibodies induced by the SLP.HIVconsv vaccines can clearly result in a positive reaction in routine HIV-1 tests. This should be explained to any future volunteers in trials of SLP.HIVconsv vaccines, should these vaccine candidates progress to clinical testing, and additional assays should be offered by the study team, which would unequivocally distinguish vaccine-elicited seropositivity from a true HIV-1 infection. On the other hand, for genetic vaccines delivering the HIVconsv immunogen, the absence of Ab induction is reassuring and will simplify any future trial procedures. These results provide guidance for informed consent procedures in volunteer recruitment for future vaccine clinical trials.

Finally, the Thai phase 3 clinical trial RV144, in which an ALVAC-HIV vCP1521Gag/Pro clade B, Env clade E prime/bivalent recombinant gp120 clade B/E boost provided a limited efficacy of 31%, has induced a resurgence of interest in HIV-1 vaccine-induced humoral responses 48. Studies of immune correlates of protection indicate that antibodies directed against the V1/V2 loops of the HIV-1 Env, including functions such as Ab-dependent cell-mediated cytotoxicity, may play a role in decreasing HIV-1 acquisition even in the absence of neutralization 28,29. Similarly, partial protection by non-neutralizing antibodies was also found following SIV challenge of rhesus monkeys vaccinated with SLP.SIVconsv derived from the simian immunodeficiency virus regions corresponding to HIVconsv 42. While HIVconsv does contain conserved regions of gp120 these are different to the region recognized by the antibodies that mediated ADCC in RV144, nevertheless it will be important to investigate whether the protective ability as well as neutralizing ability of the antibodies found following SLP.HIVconsv or SLP.SIVconsv vaccination can be further enhanced by a subsequent boost with a properly adjuvanted Env recombinant protein immunogen.

## Supporting Information

**Table S1**. HIVconsv-­specific IgM and IgG antibody titres in vaccinated animals.
