# Supplementary material for: Humoral responses to HIVconsv induced by heterologous vaccine modalities in rhesus macaques
Source: Immun Inflamm Dis. 2015 Mar 11;3(2):82–93. doi: 10.1002/iid3.52 (PMC4444151; doi:10.1002/iid3.52)
Supplement: Supplementary file 1 [file iid30003-0082-sd1.pdf]

Table S1. HIVconsv-specific IgM and IgG antibody titres in vaccinated animals

Animal	Peptide	ONE		OCTAVIA		OZONE		Peptide	ONE		OCTAVIA		OZONE	
Vaccination	Pool	IgM	IgG	IgM	IgG	IgM	IgG	Pool	IgM	IgG	IgM	IgG	IgM	IgG
SLP.HIVconsv (1)*	1	227**	50	220	50	421	199	2	194	50	183	50	445	154
SLP.HIVconsv (2)	1	407	500	236	538	375	3368	2	467	2309	220	985	581	4025
ChAdV63.HIVconsv	1	174	50	50	291	318	893	2	148	300	50	715	353	604
MVA.HIVconsv	1	68	50	50	50	151	232	2	58	50	50	50	189	271
VREP.HIVconsv	1	127	50	50	50	158	303	2	80	50	50	50	193	312
SLP.HIVconsv (1)	3	214	50	248	50	432	177	4	229	50	230	50	494	125
SLP.HIVconsv (2)	3	478	1540	263	1257	505	5222	4	465	713	295	1529	493	3073
ChAdV63.HIVconsv	3	182	189	50	684	299	638	4	185	50	68	993	317	537
MVA.HIVconsv	3	92	50	50	175	180	765	4	108	50	64	186	178	159
VREP.HIVconsv	3	110	50	50	116	134	963	4	138	50	101	117	159	183
SLP.HIVconsv (1)	5	195	50	87	50	386	284	6	205	50	143	50	436	326
SLP.HIVconsv (2)	5	462	1294	198	602	444	5760	6	513	3568	201	1343	453	6690
ChAdV63.HIVconsv	5	162	149	50	162	329	1813	6	179	822	50	894	293	1173
MVA.HIVconsv	5	65	50	50	50	159	834	6	102	135	50	123	144	677
VREP.HIVconsv	5	66	50	50	50	145	624	6	126	118	50	88	130	664

*All antibody titres in plasma samples from blood taken before SLP.HIVconsv administration were less than 50.

** Antibody titres were measured in plasma from blood taken 8-10 days after vaccination
